# Case report: A rare EBV-associated T/NK cell monomorphic posttransplant lymphoproliferative disorder

**DOI:** 10.3389/fimmu.2024.1491681

**Published:** 2024-10-24

**Authors:** Xin Jiang, Yao-Yu Zhang, Xiao-Wei Li, Xiao-Dong Li, Zhan-Yuan Li, Wen-Jun Meng, Sha-Dan Li

**Affiliations:** ^1^ Department of Urology, The General Hospital of Western Theater Command, Chengdu, China; ^2^ Department of Biotherapy, Cancer Center, West China Hospital, Sichuan University, Chengdu, China

**Keywords:** kidney transplantation, Epstein-Barr virus, case report, nursing experience, posttransplant lymphoproliferative disorder

## Abstract

**Background:**

Kidney transplantation (KT) is the best treatment for patients with end-stage renal disease. However, postoperative complications remain the main issues faced during KT recovery period. Posttransplant lymphoproliferative disorders (PTLD) are one of the severe and life-threatening complications that occur after KT while the recipient is undergoing immunosuppressive therapy. PTLD risk factors include Epstein-Barr virus (EBV) infection, the cumulative degree of immunosuppression, as well as genetic aspects. PTLD is more common in the transplanted organ itself and its surroundings, and the central nervous system, while PTLD involving the pharyngeal soft tissue is relatively rare, with only a few reported case reports. Therefore, systematic experience is scarce regarding whether the treatment or the care.

**Case presentation:**

Herein, we report a 41-year-old male, underwent a reproductive KT due to chronic renal insufficiency. Recurrent fever, pharyngeal pain, and bilateral cervical lymph node enlargement were recurred during five years’ follow-up after KT surgery. In this inpatient experience, the patient vomited a large amount of blood from the oropharynx, then the tonsil artery was ligated by emergency operation. EBV-associated T/NK cell monomorphic PTLD was eventually diagnosed by blood EBV DNA test, pharyngeal biopsy, and corresponding pathological examination. After six cycles of R-CHOP chemotherapy, the clinical symptoms and laboratory tests changed into normal. Subsequent three years’ follow-up shows no tumor recurrence and good transplant kidney function.

**Conclusion:**

This rare case report describes a manifestation of PTLD with pharyngeal involvement. Early diagnosis using histopathological examination is crucial to prevent damage to the throat and airway, and even life-threatening conditions. Discontinuing immunosuppression and starting systemic treatment can help in disease regression. Since the low incidence of this disease, limited clinical experience, and limited data, our experience with a smooth recovery through efficacy treatment and nursing can provide a reference for the development of new clinical drugs and diagnostic and treatment plans of patients with PTLD in the future.

## Introduction

Kidney transplantation (KT) is the best treatment for patients with end-stage renal disease (1). Globally, the total number of patients undergone KT is estimated to be 2.5 million by 2020 and is expected to rise to 5.4 million by 2030 (2). However, postoperative complications remain the main issues faced during KT recovery period (3). Posttransplant lymphoproliferative disorders (PTLD) are one of the severe and life-threatening complications that occur after KT while the recipient is undergoing immunosuppressive therapy (4). It includes a spectrum of lymphoproliferative diseases which are highly associated with Epstein-Barr virus (EBV) infection, typically occurring within the first year after hematopoietic stem cell or solid organ transplantation (5–7). EBV is a ubiquitous viral pathogen with a seroprevalence rate exceeding 90% in adults (6, 8). Also, this virus is a potential gamma-herpesvirus with known oncogenic potential (9). Apart from EBV infection, PTLD risk factors also involve the cumulative degree of immunosuppression, as well as genetic aspects (10). The most common cause of PTLD is EBV infection, which is also a consequence of immunosuppressive therapy (11). Most PTLDs with EBV infection originate from B-cells, although T-cell originated and EBV-negative cases also exist (12, 13). Under conditions of immunosuppression, other viruses such as Hepatitis C Virus and cytomegalovirus can also trigger PTLD (14). As a result, PTLD is a severe, life-threatening complication that can occur in organ transplant recipients during immunosuppressive therapy, reducing the survival rates of both patients and the transplanted organs (15, 16).

In 2017, the WHO defined six PTLD subtypes, divided into two major categories: non-destructive PTLD and destructive PTLD (17). Non-destructive PTLDs are early-stage diseases with mild symptoms. But the symptom of destructive PTLD varies on the basis of different cell type. The subgroup of destructive PTLD includes polymorphic PTLD (lymphocyte proliferation of B and T cells), monomorphic PTLD (diffuse large B-cell lymphoma is the most common), and Classic Hodgkin lymphoma-like PTLD. According to the lesion location inside or outside the lymph node, PTLD can also be divided into intra-nodal and extra-nodal types, with extra-nodal being more common. Extra-nodal PTLD varies by different transplanted organ, accounting for 70% of cases, but rarely involves the skin. Intra-nodal PTLD mainly manifests as lymph node enlargement, with mild to moderate uniform enhancement on enhanced computed tomography (CT), less frequent lymph node necrosis, and are more commonly seen in mediastinal and retroperitoneal lymph nodes. The affected lymph nodes lose the normal lymph node morphology such as the hilum, and can coalesce locally (6). The ear, tonsils, sinuses, salivary glands, gingiva, and cervical lymph nodes in the head and neck region may be affected by PTLD, and systemic symptoms such as febrile diseases and flu-like syndromes may also occur, while involvement of the upper respiratory tract, especially the pharyngeal area, is rarely reported (18). In fact, PTLD is more common in the transplanted organ itself and its surroundings, and the central nervous system (19). Only a few PTLD cases were reported which involved the pharyngeal soft tissue (19). Therefore, systematic experience is scarce regarding whether the treatment or the care.

Herein, we report a 41-year-old KT patient diagnosed as PTLD but very rarely occurred in the pharynx. Meanwhile, the successful nursing experience on this patient is concurrently discussed.

## Case presentation

**Figure 1:**
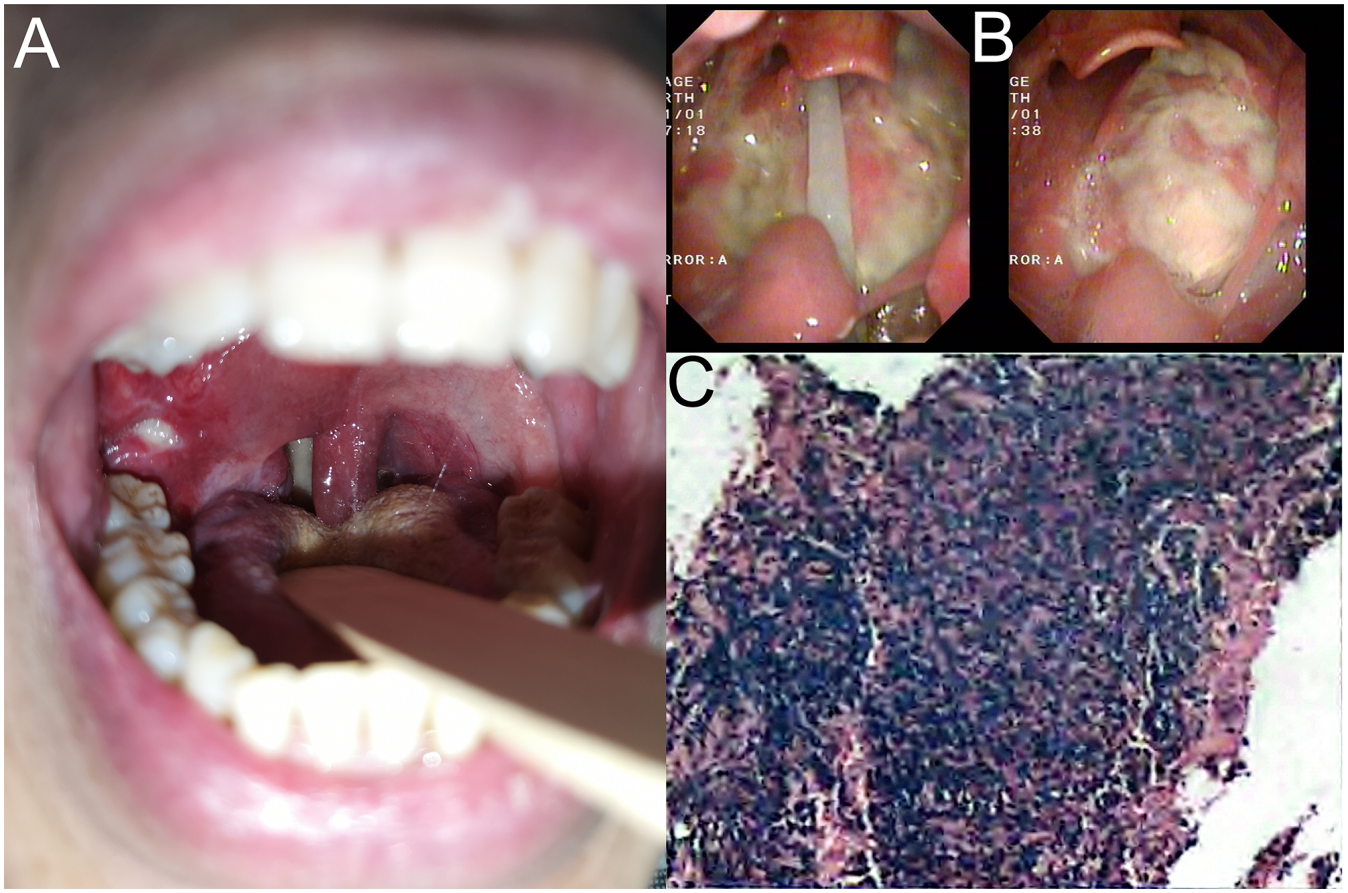
**(A)** Neoplasm was found in the pharynx on November 19, 2020, considering common mucosal ulcers. **(B)** Pharyngeal tissue biopsy displayed the condition of the oropharynx. **(C)** Pharyngeal tissue biopsy revealed inflammatory and necrotic tissue by pathological examination.

A 41-year-old male, underwent a reproductive KT due to chronic renal insufficiency (uremia stage) on August 13, 2015, and has been on long-term immunosuppressive therapy with mycophenolate mofetil, tacrolimus, and prednisone. The patient had recurrent fever, pharyngeal pain, and bilateral cervical lymph node enlargement after KT surgery. The patient had three consecutive positive tests for EBV DNA in whole blood from March to April 2019, which turned negative after treatment with valacyclovir. On November 19, 2020, neoplasm was found in the pharynx during routine follow-up, considering common mucosal ulcers (**Figure 1A**). On January 16, 2021, the patient was admitted to our hospital again (The General Hospital of Western Theater Command, Chengdu, China) with recurrent fever, cough, and expectoration for three days. The patient reported that the sputum was scanty, viscous, and difficult to expectorate, accompanied by fatigue, chest tightness, and discomfort in the throat and pharynx. Physical examination revealed swelling and ulceration of the oropharynx and right tonsil, with purulent secretion exudation. Bilateral cervical lymph nodes were palpable and enlarged, with the largest diameter being approximately 1.0cm × 1.0cm, firm and fixed, without tenderness.

**Figure 2:**
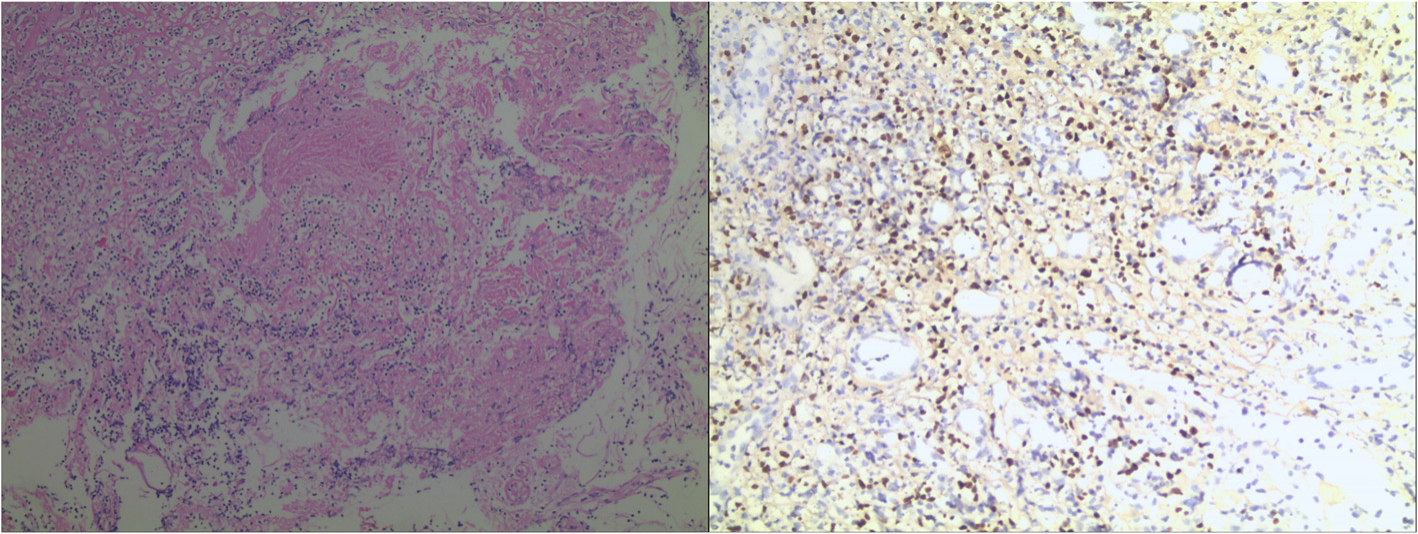
Hematoxylin-eosin staining after pharyngeal biopsy revealed the immunostaining positive for CD3 and CD56, and high proliferation activity (Ki-67 about 60%).

Upon admission, the patient’s whole blood EBV DNA test was positive, suggesting EBV-associated PTLD. The patient underwent several times of pharyngeal tissue biopsy (**Figure 1B**), all of which showed inflammatory and necrotic tissue by pathological examination (**Figure 1C**). During the treatment, the patient vomited a large amount of blood from the oropharynx, followed by a drop in blood pressure. An emergency neck vein channel was established for rapid fluid replenishment and blood transfusion, and the patient was successfully resuscitated after cardiopulmonary resuscitation. On March 21, 2021, the patient was transferred to the intensive care unit for continued observation and underwent an emergency exploration of the oropharynx, where active bleeding from the right tonsil artery was found. We immediately ligated the tonsil artery. Subsequently, postoperative laryngoscopy and biopsies of the oropharynx, tonsils, and cervical lymph nodes were performed. The pharyngeal biopsy showed a small amount of atypical lymphocytes in the necrotic tissue, with immunostaining positive for CD3 and CD56, high proliferation activity (Ki-67 about 60%), and EBER *in situ* hybridization was diffusely positive, while other markers were negative or focally positive (CD20, CD21, Pax-5, CD4, CD8, and CD30), consistent with T/NK cell monomorphic PTLD (**Figure 2**).

**Figure 3:**
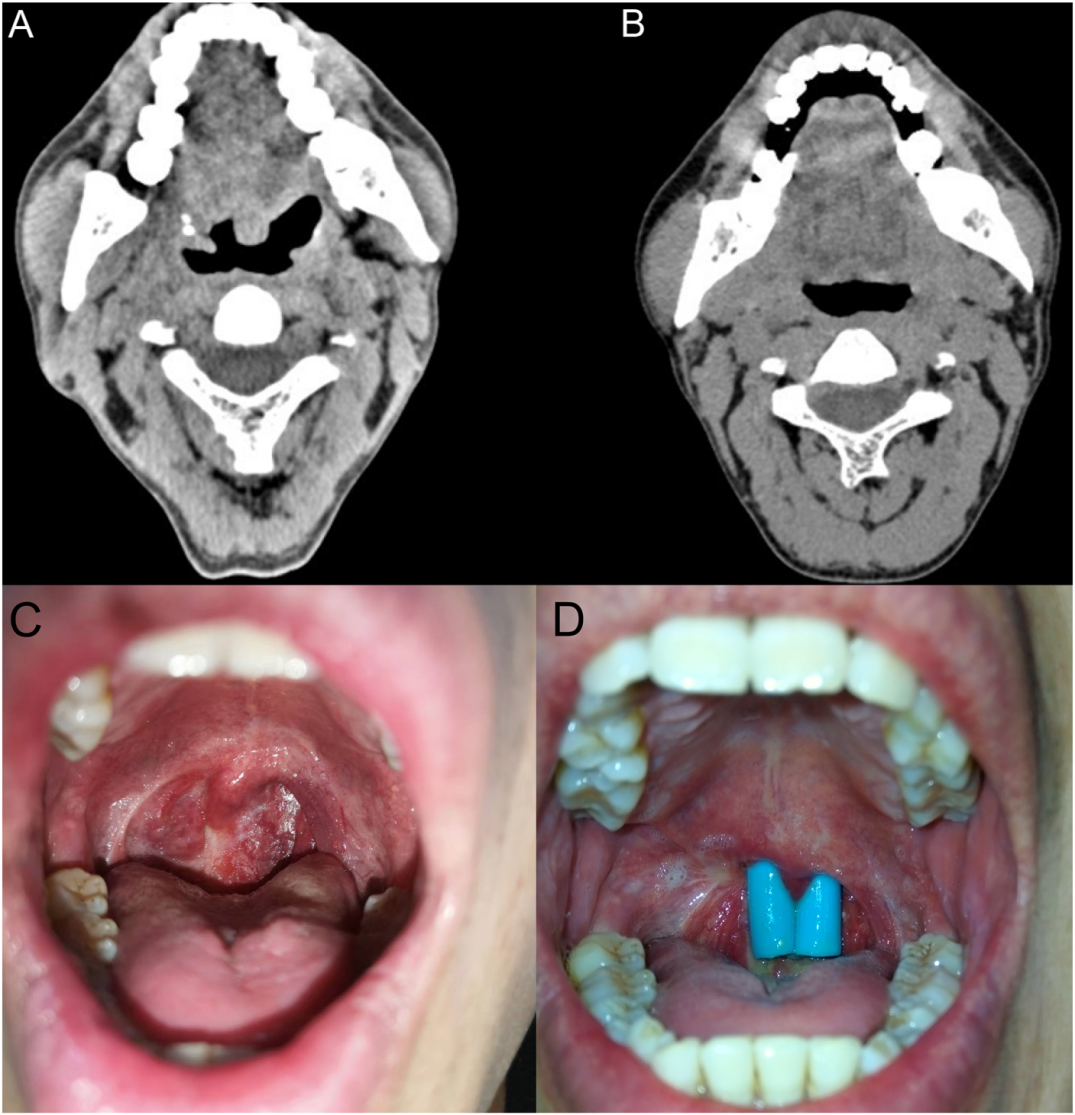
**(A)** The imaging examination prior six cycles’ R-CHOP chemotherapy on January 19, 2019. **(B)** The imaging examination post chemotherapy on February 26, 2022. **(C)** The condition of nasopharyngeal closure in the oropharynx, and nasopharyngeal closure was discovered in January 2022. **(D)** Post nasopharyngeal plasty surgery in May 2022.

**Figure 4:**
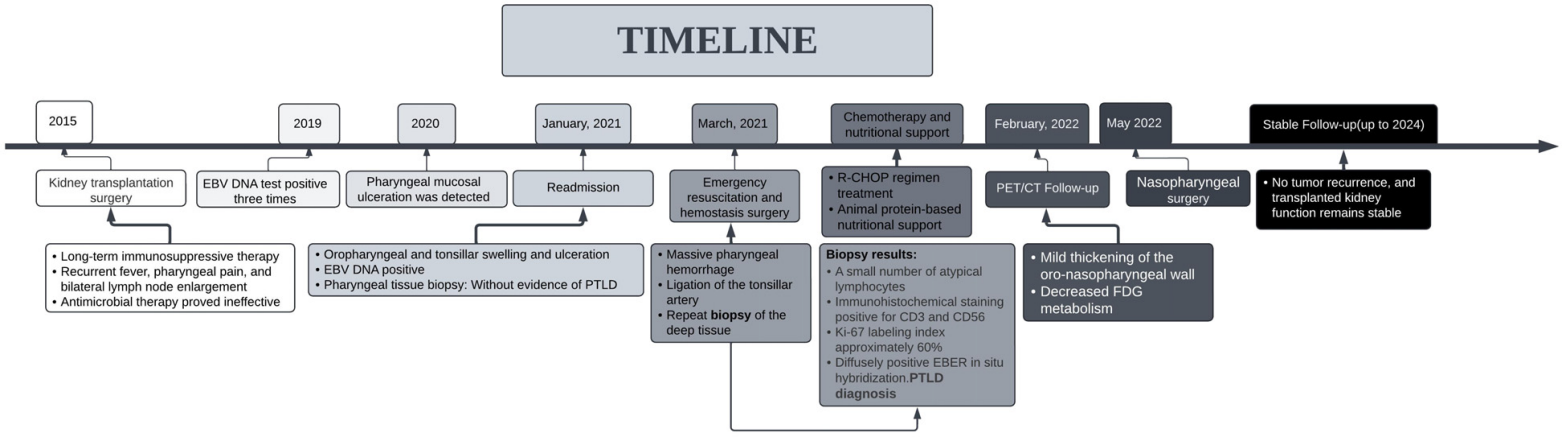
Timeline of the treatment process and clinical findings.

The diagnosis of PTLD mainly depends on clinical manifestations, peripheral blood EBV load, and pathological results. After the physical condition was improved, the patient was about to start systemic therapy. Due to the poor prognosis in the single use of rituximab for PTLD, we recommended the patient the regimen of rituximab combined with cyclophosphamide, doxorubicin, vincristine, and prednisone (R-CHOP) (20). After six cycles of R-CHOP regimen, the cervical lymph nodes regressed, and the patient had no discomfort in the throat, no fever, cough, or expectoration; blood routine and EBV DNA were normal. On February 26, 2022, a confirmation by positron emission tomography/computed tomography (PET/CT) showed slight thickening of the right wall of the nasopharynx and oropharynx, with no increased fluorodeoxyglucose (FDG) metabolism; diffuse mild increase in FDG metabolism in the red bone marrow distribution area, with reduced FDG metabolism compared to the previous PET/CT (**Figures 3A, 3B**). The patient underwent nasopharyngeal plasty in May 2022 due to stenosis caused by adhesion of the soft palate and the posterior pharyngeal wall, leading to nasopharyngeal closure (**Figures 3C, 3D**). The patient has been followed up for three years, with no tumor recurrence and good transplant kidney function. Also, a timeline is displayed to show the development of these treatment process and clinical findings (**Figure 4**).

## Discussion

In this study, we reported a rare EBV-associated T/NK cell monomorphic PTLD. It is generally accepted that two major factors are associated with the occurrence of PTLD: the serological status of EBV in the recipient and donor; and the depth of T-cell immunosuppression before and after transplantation (9). In fact, PTLD is associated with EBV infection in up to 80% of cases of hematopoietic stem cell transplantation and solid organ transplantation. The common sites of PTLD include lymph nodes, gastrointestinal tract, central nervous system, subcutaneous tissue, lungs, liver, kidneys, etc. (21), while symptoms and clinical manifestations in the ear, nose, and throat often occur in the early stage of PTLD (22). In our KT case, due to recurrent fever, pharyngeal and tonsillar enlargement, and ulceration with purulent exudate, we initially diagnosed acute tonsillitis. The treatment effect was poor after repeated anti-infection treatments. After a positive EBV test in the whole blood, these combining clues strongly suggested PTLD associated with EBV infection. However, after consultation with our otolaryngologist and twice pathological biopsies, PTLD was not indicated, so effective treatment could not be taken, leading to a delay in the disease for several months. It was not until the oropharyngeal ulceration involved the tonsillar artery, causing fatal massive bleeding and suffocation, that the patient underwent emergency rescue and surgery for hemostasis and was sent to the intensive care unit. After another consultation with our experienced otolaryngologist, it was concluded that this is a very rare case. It is possible that the tissue biopsies taken in the first two attempts were too shallow and did not obtain positive pathological tissue. So the third time, the head of our otolaryngology department personally operated, increasing the depth and volume of tissue excision, which finally led to a definitive PTLD diagnosis and effective treatment. Therefore, routine and repeating pathological biopsy and immunological testing of the patient’s oropharyngeal and tonsillar tissue were necessarily performed. Finally, the diagnosis of PTLD mainly relies on clinical manifestations, peripheral blood EBV load, and repeated pathological results.

Studies have shown that closely monitoring EBV DNA levels after transplantation can help identify patients at risk of chronic high load of EBV and PTLD (23). Early primary EBV infection is a risk for chronic EBV-related diseases, so avoiding primary infection after transplantation is important. For KT recipients with unexplained fever and elevated serum creatinine, active infections of cytomegalovirus and EBV generally exist (24). Before diagnosing PTLD, it is important to exclude specific and non-specific lymphoplasmacellular infiltration related to infection, graft rejection, graft-versus-host disease, or recurrence of known lymphoma before transplantation (25). If the organ donor is EBV seropositive, PTLD should be considered in time when the diagnosis is unclear. There have also been reports of cases of EBV and herpes virus-related hemophagocytic syndrome after KT (26). For high-risk PTLD recipients, regular monitoring of EBV viral load is warranted. Studies have confirmed that a preemptive treatment strategy when monitoring viral load is elevated can reduce the incidence of PTLD (27). Due to the expression of EBV antigens, immunotherapy is an effective treatment strategy (28). It may also reduce the occurrence of PTLD by decreasing the dose of immunosuppressants and prophylactic use of acyclovir and ganciclovir for antiviral treatment (29, 30). Additionally, the prevention of PTLD is also very important, by gradually reducing immunosuppression, using mTOR inhibitors instead of calcineurin inhibitors, and regular monitoring of EBV load, especially in EBV-mismatched patients (31).

Except for the prevention and treatment of PTLD, the nursing care is a key point during the disease development. Among all KT patients, 87% of them have one or more issues related to physical or mental health, 55% suffered decreased muscle strength or walking ability, 45% have poor nutritional status, and 36% exhibit high anxiety or depression (32). As a result, the compliance of KT patients is relatively poor (33). Due to the rarity of T/NK cell monomorphic PTLD and its hidden clinical symptoms, our patient was prone to further anxiety, unease, and fear. Therefore, it is essential for nursing staff to actively communicate with the patient, patiently soothe the patient’s negative emotions, encourage and support the patient, provide psychological counseling, and enhance the patient’s confidence in overcoming the disease. At the same time, it is also important to pacify the patient’s family to reduce the patient’s psychological and spiritual burden and improve patient compliance and cooperation.

In our case, the patient has been on long-term immunosuppressive therapy with immunosuppressants and hormones after KT, in a state of low immunity. To maintain the normal function of the transplanted kidney, the patient’s daily diet must be carefully considered to avoid affecting the concentration of immunosuppressants. After the diagnosis of T/NK cell monomorphic PTLD, nutritional supplementation is needed to balance the nutritional loss during anti-tumor treatment. Traditional views suggest that animal protein, which is high in essential amino acids, is beneficial to kidney function; while plant protein, especially soy protein, may increase the burden on the kidneys. When formulating a nutritional supplement plan for the patient, animal protein should be prioritized.

KT recipients, due to postoperative immunosuppressive therapy, are at high risk of viral infections as their cellular immunity is strongly suppressed. Post-transplant EBV infection can lead to PTLD and severe pneumonia. Although the incidence of PTLD is low, the disease progresses rapidly and has a high mortality rate, lacking comparative data to assess potential treatment strategies (34). It is recommended that clinicians increase their awareness of the risk of PTLD and conduct regular EBV load screening in EBV seronegative recipients receiving EBV seropositive grafts, and actively adopt effective preventive measures and treatment plans in the first few years after KT, which will help improve patient outcomes (8, 35).

Our study reported a rare case of EBV-associated T/NK cell monomorphic PTLD, adding new insights to the literature. However, it has certain limitations, including its single-case focus, which restricts generalizability due to a lack of large-scale, multicenter studies. Current diagnostic methods do not fully capture PTLD complexity; for example, EBV DNA detection may not distinguish between infection and real PTLD. Also, the histopathological evaluation is subject to sampling errors, and the three-year follow-up may not suffice for assessing long-term outcomes. The retrospective design limits data control, and the absence of standardized metrics may introduce bias in evaluating treatment efficacy. Despite these issues, the study enhances understanding of EBV-associated PTLD and sets the stage for future research to address these limitations.

## Conclusion

In conclusion, this rare case report describes a manifestation of PTLD with pharyngeal involvement. Early and multiple diagnostic methods, especially histopathological examination, are crucial to prevent the risk of acute oropharyngeal bleeding and suffocation, and prevent damage to the throat and airway, which are severe life-threatening conditions. Our clinical experience can also help reduce treatment time, and decreasing treatment-related costs when similar rare condition occurs, which has significant clinical reference value. Moreover, discontinuing immunosuppression and starting systemic treatment can help in disease regression. Since the low incidence of this disease, limited clinical experience, and limited data, our experience with a smooth recovery through efficacy treatment and nursing can provide a reference for the development of new clinical drugs and diagnostic and treatment plans of patients with PTLD in the future.

## Data Availability

The original contributions presented in the study are included in the article/supplementary material. Further inquiries can be directed to the corresponding author/s.
